# Antagonism of Phthesis and Intermittent Fever

**Published:** 1844-02

**Authors:** 


*Antagonism of Phthisis and Intermittent Fever.*—No such antagonism has been observed by the profession of the United States; but, on the contrary, it is found that the persistence of ague is apt to lead to the development of tubercles in the lungs. Dr. Richardson's paper, in this number, may be referred to for testimony. The following is to the same point:

"Dr. Gouzée, chief physician to the Military Hospital, Antwerp, in a note addressed to the editor of the *Gazette Medicale*, says, 'The partizans of this opinion affirm that phthisis is unknown in countries where marsh miasma reigns.' This may be true with some, but certainly is not the case at Antwerp, where, though intermittent fever is endemical, phthisis is very prevalent. Thus, from 1834 to 1842, of 453 deaths at the Military Hospital, 123 were caused by phthisis, or one in three and two-thirds. During the first six months of 1843, of 17 deaths, 9 were from phthisis, or more than one-half, though intermittent fever was more prevalent than usual. Moreover, no doubt can exist as to the nature of the disease, the *post mortem* examination having demonstrated the tubercles. The same remark is applicable to the inhabitants of the town; therefore, the law announced by Dr. Boudin is not without an exception; for, in Antwerp, phthisis and intermittent fever reign simultaneously, and the one does not prevent the development of the other."—*Medical Times*, Dec. 1843.

